# Development, Validation, and Deployment of a Time-Dependent Machine Learning Model for Predicting One-Year Mortality Risk in Critically Ill Patients with Heart Failure

**DOI:** 10.3390/bioengineering12050511

**Published:** 2025-05-12

**Authors:** Jiuyi Wang, Qingxia Kang, Shiqi Tian, Shunli Zhang, Kai Wang, Guibo Feng

**Affiliations:** 1 Department of General Medicine, The Affiliated Yongchuan Hospital of Chongqing Medical University, Chongqing 402160, China; 2023420066@stu.cqmu.edu.cn (J.W.); 2024121535@stu.cqmu.edu.cn (S.T.); zsl2566@163.com (S.Z.); 2 Department of Cardiology, The Affiliated Yongchuan Hospital of Chongqing Medical University, Chongqing 402160, China; kqxychcqmu@163.com; 3 Department of Cardiology, The Second Affiliated Hospital of Chongqing Medical University, Chongqing 401336, China

**Keywords:** heart failure, intensive care unit, machine learning, time-dependent, random survival forest, XGBoost, MIMIC-IV database

## Abstract

**Background:** Heart failure (HF) ranks among the foremost causes of mortality globally, exhibiting particularly high prevalence and significant impact within intensive care units (ICUs). This study sought to develop, validate, and deploy a time-dependent machine learning model aimed at predicting the one-year all-cause mortality risk in ICU patients diagnosed with HF, thereby facilitating precise prognostic evaluation and risk stratification. **Methods:** This study encompassed a cohort of 8960 ICU patients with HF sourced from the Medical Information Mart for Intensive Care IV (MIMIC-IV) database (version 3.1). This latest version of the database added data from 2020 to 2022 on the basis of version 2.2 (covering data from 2008 to 2019); therefore, data spanning 2008 to 2019 (*n* = 5748) were designated for the training set, while data from 2020 to 2022 (*n* = 3212) were reserved for the test set. The primary endpoint of interest was one-year all-cause mortality. Least Absolute Shrinkage and Selection Operator (LASSO) regression was employed to select predictive features from an initial pool of 64 candidate variables (including demographic characteristics, vital signs, comorbidities and complications, therapeutic interventions, routine laboratory data, and disease severity scores). Four predictive models were developed and compared: Cox proportional hazards, random survival forest (RSF), Cox proportional hazards deep neural network (DeepSurv), and eXtreme Gradient Boosting (XGBoost). Model performance was assessed using the concordance index (C-index) and Brier score, with model interpretability addressed through SHapley Additive exPlanations (SHAP) and time-dependent Survival SHapley Additive exPlanations (SurvSHAP(t)). **Results:** This study revealed a one-year mortality rate of 46.1% within the population under investigation. In the training set, LASSO effectively identified 24 features in the model. In the test set, the XGBoost model exhibited superior predictive performance, as evidenced by a C-index of 0.772 and a Brier score of 0.161, outperforming the Cox model (C-index: 0.740, Brier score: 0.175), the RSF model (C-index: 0.747, Brier score: 0.178), and the DeepSur model (C-index: 0.723, Brier score: 0.183). Decision curve analysis validated the clinical utility of the XGBoost model across a broad spectrum of risk thresholds. Feature importance analysis identified the red cell distribution width-to-albumin ratio (RAR), Charlson Comorbidity Index, Simplified Acute Physiology Score II (SAPS II), Acute Physiology Score III (APS III), and the age–bilirubin–INR–creatinine (ABIC) score as the top five predictive factors. Consequently, an online risk prediction tool based on this model has been developed and is publicly accessible. **Conclusions:** The time-dependent XGBoost model demonstrated robust predictive capability in evaluating the one-year all-cause mortality risk in critically ill HF patients. This model offered a useful tool for early risk identification and supported timely interventions.

## 1. Introduction

Heart failure (HF) remains a major and global health issue, marked by persistently high morbidity and mortality rates [1]. From 1990 to 2019, the global prevalence of HF increased by 106.3%, reaching 56.2 million cases [2]. The complex interplay of comorbidities, HF severity, and multi-organ dysfunction necessitates advanced care for critically ill heart failure patients in the intensive care unit (ICU), presenting substantial challenges for comprehensive management. Acute HF signifies a critical juncture in disease progression, as ICU survivors experience a one-year mortality rate of 46.5%, a rate comparable to or higher than that observed in many malignancies [3,4]. Accurate prediction of mortality risk in these patients is essential for guiding clinical decision-making, optimizing resource allocation, and tailoring therapeutic strategies. Although established prognostic tools such as the Seattle Heart Failure Model (SHFM) [5], Get With The Guidelines-Heart Failure (GWTG-HF) [6], and AHEAD (A: atrial fibrillation; H: hemoglobin; E: elderly; A: abnormal renal parameters; and D: diabetes mellitus) score [7] are widely used, their reliance on linear statistical assumptions limits their capacity to capture the complex interactions among clinical variables, thereby constraining predictive accuracy in heterogeneous ICU populations.

Recent advancements in machine learning (ML) present transformative potential for prognostic modeling by excelling in the processing of high-dimensional data and the identification of nonlinear relationships. Firstly, ensemble algorithms (such as eXtreme Gradient Boosting (XGBoost) and Light Gradient Boosting Machine (LightGBM)) effectively decode nonlinear biomarker interactions; for instance, XGBoost has demonstrated superior performance compared to logistic regression in distinguishing one-year mortality [8]. Secondly, interpretability frameworks address the challenges posed by black box models; most physicians preferred ML outputs that were augmented with model-agnostic interpretability methods, with significant correlations observed between clinician comprehension and interpretability, as well as between interpretability and trust [9]. Although ML has shown superior performance over traditional methods in predicting heart failure outcomes, critical gaps remain. Specifically, current ML applications often treat mortality as a binary outcome, neglecting the time-dependent survival information [10,11,12]. Concurrently, validated composite laboratory indices—such as albumin-corrected anion gap (ACAG) [13] and albumin–bilirubin index (ALBI) [14], and red cell distribution width-to-albumin ratio (RAR) [15]—were underutilized in ML frameworks, despite their established prognostic value.

To address these gaps, we developed and validated a time-dependent ML model to predict one-year all-cause mortality in ICU-admitted heart failure patients, utilizing the Medical Information Mart for Intensive Care IV database (MIMIC-IV version 3.1, https://physionet.org/content/mimiciv/3.1/, accessed on 24 November 2024) [16]. Our approach uniquely integrates routine clinical parameters with validated prognostic composites while employing advanced techniques for model interpretation, validation, and online deployment. We developed the first time-dependent machine learning model specifically for critically ill heart failure patients in the ICU setting that is capable of providing dynamic risk predictions across the 365-day post-discharge timeline. Our approach integrates novel composite indices (such as RAR and ABIC) with traditional prognostic scores, enhancing predictive accuracy beyond what either approach can achieve independently. By implementing advanced interpretability techniques (SurvSHAP(t)), we provide time-dependent feature importance analysis, offering insights into how predictors’ influence changes over the follow-up period. To translate these complex models into clinical practice, we deployed a free, web-based calculator that serves as an accessible decision support tool for bedside use. Notably, our model demonstrates generalizability across the COVID-19 pandemic period, suggesting robust performance despite significant healthcare disruption. Our key findings reveal that the XGBoost-based model achieved superior performance compared to traditional approaches, with novel laboratory indices and composite scores emerging as the most influential predictors.

The remainder of this manuscript is organized as follows: In Section 2, we describe our study population, data collection procedures, feature extraction, and the development of four different machine learning models. Section 3 presents the baseline characteristics of our study cohort, the performance metrics of the various models, feature importance analysis, and details of our web-based calculator deployment. In Section 4, we contextualize our findings within the existing literature, explore the clinical implications of our model, acknowledge limitations, and suggest directions for future research. Finally, Section 5 summarizes the key findings and potential impact of our work. The Supplementary Materials provide additional methodological details and supplementary analyses.

## 2. Materials and Methods

### 2.1. Research Problem and Objectives

This study addresses the critical research problem of accurately predicting one-year mortality risk in critically ill heart failure patients following ICU admission. Specifically, we aimed to develop a time-dependent machine learning model that can (1) predict one-year all-cause mortality risk dynamically, enabling survival probability estimation at any time point within 365 days post-discharge; (2) integrate composite biomarkers (e.g., RAR, ABIC) with routine clinical parameters to enhance predictive accuracy and pathophysiological relevance; and (3) validate the model’s clinical utility through rigorous temporal calibration, interpretability frameworks (SHAP/SurvSHAP(t)), and deployment as an open-access tool.

### 2.2. Sample Size and Study Population

**Figure 1:**
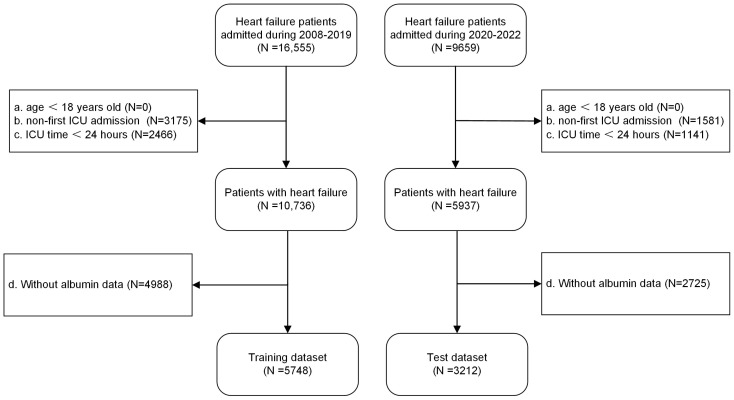
Flow chart of study population inclusion.

This retrospective cohort study utilized the MIMIC-IV database (version 3.1), a comprehensive and publicly accessible critical care repository containing detailed clinical data from critically ill patients treated at Beth Israel Deaconess Medical Center between 2008 and 2022. Study participants were selected based on predefined inclusion and exclusion criteria, with inclusion requiring a definitive diagnosis of congestive HF (International Classification of Diseases, 9th Revision [ICD-9] code 428* and 10th Revision [ICD-10] code I50*). Exclusion criteria comprised (1) age < 18 years; (2) no prior ICU admission history; (3) ICU length of stay < 24 h; and (4) missing albumin measurements. The patient selection flowchart outlined the screening process, resulting in a final analytical cohort of 8960 patients (Figure 1).

This study strictly adhered to ethical guidelines established by institutional review boards, national research committees, and the 1964 Declaration of Helsinki and its subsequent amendments. Informed consent was waived as the research involved secondary analysis of de-identified public data without direct or indirect identifiers. Furthermore, no additional ethical review was required given the use of publicly available datasets.

### 2.3. Data Collection and Outcome Definition

Within 24 h of ICU admission, the initial measurement was used to extract clinical data. Data points with over 20% missing values were excluded. To tackle the problem of incomplete data, the multiple imputation technique was applied based on the missing-at-random (MAR) assumption. In particular, variables with less than 20% missing information underwent multiple imputation through chained equations (MICE) with the help of the mice package, generating five complete datasets over 50 iterations, after which Rubin’s rules were applied for pooled estimation.

Clinical data were systematically extracted according to a predefined protocol, encompassing demographic characteristics (age, sex), vital signs (heart rate, respiratory rate, oxygen saturation (SpO_2_), body temperature, systolic and diastolic blood pressure), comorbidities and complications (diabetes mellitus, hypertension, chronic kidney disease (CKD), chronic obstructive pulmonary disease (COPD), acute kidney injury (AKI) and AKI staging, and sepsis), and cardiac function parameter (left ventricular ejection fraction (LVEF)). Therapeutic interventions were categorized as continuous renal replacement therapy (CRRT) or mechanical ventilation. Disease severity was evaluated using multiple scoring systems, including Sequential Organ Failure Assessment (SOFA), Acute Physiology Score III (APS III), Acute Physiology and Chronic Health Evaluation (APACHE) III, Glasgow Coma Scale (GCS), Simplified Acute Physiology Score II (SAPS II), Charlson Comorbidity Index (CCI), Systemic Inflammatory Response Syndrome (SIRS) score, and Oxford Acute Severity of Illness Score (OASIS).

Laboratory parameters included complete blood count indices (white blood cell count (WBC), red blood cell count (RBC), hemoglobin, hematocrit, platelet count, erythrocyte indices (mean corpuscular volume (MCV), mean corpuscular hemoglobin (MCH), mean corpuscular hemoglobin concentration (MCHC), and red cell distribution width (RDW)), leukocyte differentials (neutrophils, lymphocytes, basophils, and eosinophils), liver function tests (alanine aminotransferase (ALT), aspartate aminotransferase (AST), albumin, and total bilirubin), renal function markers (creatinine, blood urea nitrogen (BUN)), serum glucose, electrolyte and acid-base balance parameters (sodium, calcium, chloride, and potassium), coagulation profiles (international normalized ratio (INR), prothrombin time (PT), activated partial thromboplastin time (PTT)), and arterial blood gas measurements (pH, partial pressure of carbon dioxide (PCO_2_), partial pressure of oxygen (PO_2_), lactate, and anion gap).

In alignment with published prognostic evidence, validated composite indices were calculated, including RAR, hemoglobin-to-RDW ratio (HRR), BUN-to-creatinine ratio (BCR), BUN-to-albumin ratio (BAR), albumin-to-creatinine ratio (ACR), creatinine-to-total bilirubin ratio, ALT-to-AST ratio, ACAG, ALBI, lactate-to-albumin ratio, sepsis-induced coagulopathy (SIC) score, and the age–bilirubin–INR–creatinine (ABIC) score.

The primary endpoint was defined as all-cause mortality within one year post-discharge. All parameters, including their scales (integer or interval values) and measurement units, were fully detailed in Table S1.

### 2.4. Statistical Analysis

#### 2.4.1. Data Preprocessing

The analytical matrix was developed from an initial set of 64 clinical predictor variables, with standardized preprocessing procedures implemented to ensure data quality. Continuous variables were normalized to a range of [0, 1] using min–max scaling to mitigate the impact of scale differences on ML algorithms. Categorical variables were converted into orthogonal dummy variables through one-hot encoding. Under the assumption of missing-at-random (MAR) data, variables with ≤20% missingness underwent multiple imputation via chained equations (MICE) using the mice package, generating five complete datasets through 50 iterations, followed by Rubin’s rule for pooled estimates [17].

Continuous variables were reported as medians with interquartile ranges, and between-group comparisons were conducted using Mann–Whitney U tests. Categorical variables were presented as counts and percentages, with analyses performed using chi-square tests. The two-tailed *p* value < 0.05 was considered statistically significant. All statistical analyses were conducted using R version 4.3.1 (R Foundation for Statistical Computing, Vienna, Austria, https://www.R-project.org/, accessed on 17 September 2022).

#### 2.4.2. Model Development and Evaluation

Sample size estimation was guided by the four-step predictive model methodology proposed by Riley et al. [18], and the estimated sample size was compared against the actual cohort size. Patients admitted between 2008 and 2019 were assigned to the training set, while those admitted from 2020 to 2022 constituted the temporally isolated test set. This chronological partitioning was employed to maintain the independence of the test set, thereby reducing the risk of overfitting and enhancing calibration stability during external validation.

In the training cohort, global feature selection was achieved using five-fold cross-validated Least Absolute Shrinkage and Selection Operator (LASSO) regression. Four ML models—Cox proportional hazards regression, random survival forest (RSF), eXtreme Gradient Boosting (XGBoost), and Cox proportional hazards deep neural network (DeepSurv)—were developed utilizing features selected through LASSO to predict the study endpoint. Hyperparameter optimization was conducted using five-fold cross-validation combined with Bayesian optimization.

In the test cohort, model discrimination was evaluated using Harrell’s concordance index (C-index) and the integrated cumulative/dynamic area under the receiver operating characteristic curve (C/D AUC). Model calibration was assessed via Brier scores and calibration plots, while clinical utility was examined through decision curve analysis (DCA) and clinical impact curve (CIC). The optimal model was determined based on the highest C-index and the lowest Brier score.

#### 2.4.3. Model Interpretation

To address the inherent “black box” nature of ML models, interpretability frameworks were systematically applied. Global explanations were provided using SHapley Additive exPlanations (SHAP) analysis, time-dependent variable importance metrics, and partial dependence plots.

Local interpretability for individual predictions was achieved through SurvLIME and time-dependent Survival SHapley Additive Explanations (SurvSHAP(t)), which quantified feature contributions to risk predictions across both temporal and population dimensions.

#### 2.4.4. Model Deployment

To advance clinical translation, an online risk prediction calculator was developed utilizing the R Shiny framework. This tool integrates an optimized model, allowing for the real-time generation of individualized mortality risk predictions based on clinician-provided parameters, thereby offering intuitive, data-driven support for clinical decision-making.

## 3. Results

### 3.1. Sample Size and Baseline Characteristics

The minimum required sample size was calculated to be 1922 under predefined parameters (Cox–Snell R-squared = 0.26, event rate = 45.32%, mean follow-up = 0.63 years, and 64 candidate predictors), ensuring sufficient statistical power. This study ultimately included 5748 HF patients admitted between 2008 and 2019, and 3212 patients from 2020 to 2022, thereby exceeding the minimum requirement by more than threefold (Supplementary Materials, Table S1).

Baseline characteristics indicated comparable demographics between the datasets. In the training and test sets, median age was 72.54 years and 72.42 years, with female predominance of 56.3% and 57.2%. Prevalence of comorbidities and complications included diabetes (21.3% and 19.5%), hypertension (37.2% and 34.1%), CKD (23.7% and 21.3%), and AKI (79.9% and 63.9%). Mechanical ventilation was required in 87.5% and 75.7%. The primary endpoint (one-year mortality) occurred in 45.3% (2605/5748) of the training set and 47.6% (1530/3212) of the test set, with no significant temporal discrepancies in event rates (*p* = 0.120).

### 3.2. Feature Selection and Model Development

**Figure 2:**
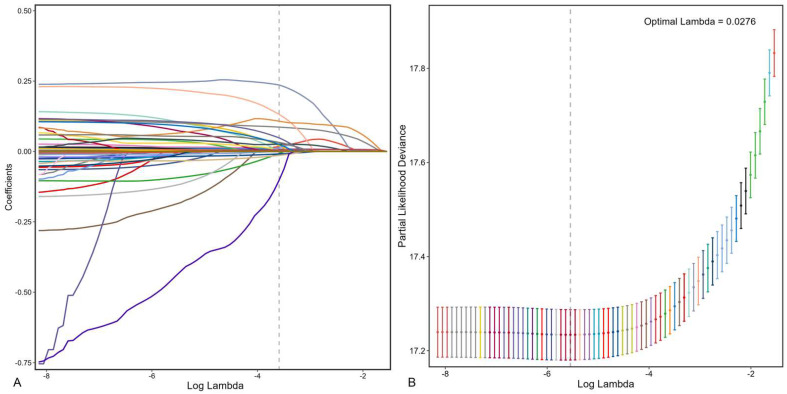
Feature selection process. Automated feature selection for 64 clinical factors was performed using Least Absolute Shrinkage and Selection Operator, which minimized the loss function binomial deviance, shrank coefficients, and produced some coefficients that are zero, allowing efficient feature selection (**A**). The algorithm outputted 24 filtered features with non-zero coefficients that were included in model generation subsequently (**B**).

In the training set, LASSO regression was employed for automated feature selection. By adjusting the regularization coefficient lambda (λ) to minimize the loss function (binomial deviance), LASSO regression produced sparse coefficients, ultimately selecting 24 out of 64 features for inclusion in the ML models. These features were identified at an optimal shrinkage parameter (λ_1se_) of 0.0276 (Figure 2).

Four ML models were developed utilizing these 24 features. Through the five-fold cross-validation, the optimized hyperparameters for each model are detailed in Table 1. Following hyperparameter optimization, the models were retrained on the complete training dataset.

### 3.3. Model Evaluation

**Figure 3:**
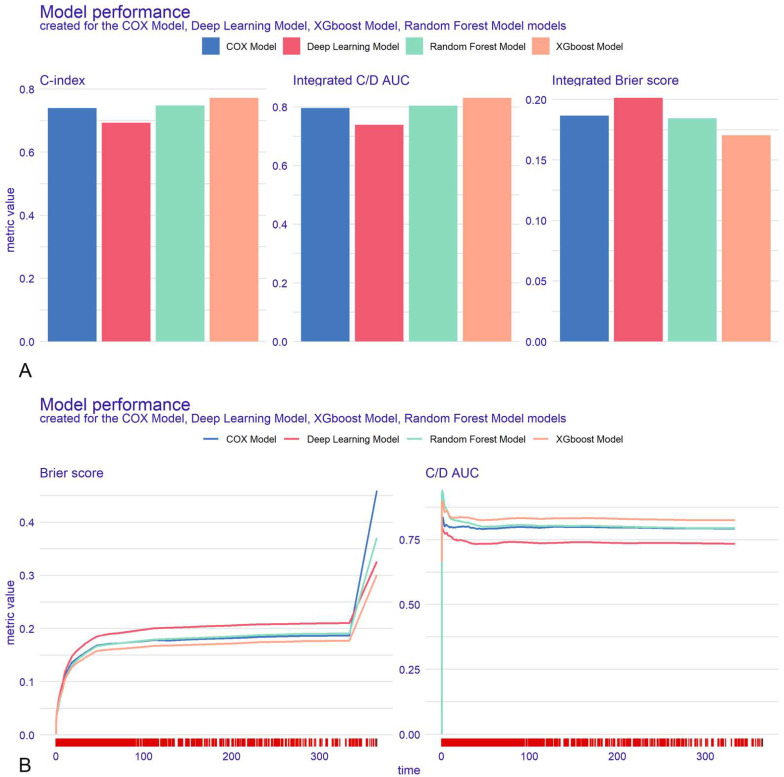
Model performance for the whole cohort. Explainable machine learning (XAI) data are shown as bar plots (**A**). Explainable machine learning (XAI) was used as a time-dependent estimation (**B**).

A comprehensive evaluation was conducted on the test cohort using metrics including the C-index, Brier score, recall rate, and D-calibration (Supplementary Materials, Figure S1). The XGBoost model exhibited superior discriminative performance, achieving a C-index of 0.772, surpassing DeepSurv (0.714), Cox regression (0.740), and RSF (0.748). XGBoost consistently outperformed the other models across secondary metrics (Figure 3A) and throughout the follow-up period (Figure 3B).

Calibration analysis indicated a strong concordance between predicted and observed mortality probabilities across all four models (Supplementary Materials, Figure S2). The XGBoost model demonstrated superior calibration performance, with a Brier score of 0.165, compared to DeepSurv (0.190), Cox regression (0.175), and RSF (0.177).

In addition, DCA indicated that the XGBoost model provided a greater net benefit compared to both the “zero mortality risk” and “all mortality” strategies, surpassing the performance of the other three models across threshold probabilities ranging from 30% to 100% (Supplementary Materials, Figure S3A). The CIC analysis further demonstrated that within this threshold range, the XGBoost model significantly minimized unnecessary interventions while enhancing risk-avoidance efficacy, thereby exhibiting superior overall intervention efficiency relative to other models (Supplementary Materials, Figure S3B). Consequently, following a comprehensive evaluation of all metrics, the XGBoost model was identified as the optimal model.

### 3.4. Model Interpretation and Online Deployment

#### 3.4.1. Global Explanations

**Figure 4:**
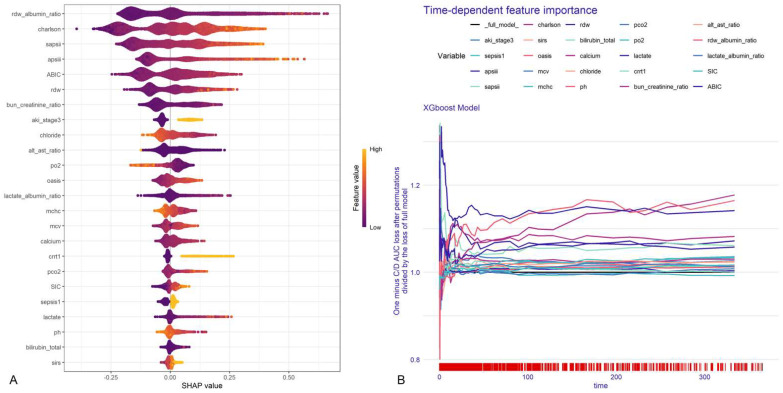
Chart of feature importance ranking in test data (**A**). The importance ranking of the 24 risk factors with stability and interpretation using the optimal model. Each point in the graph represents the SHAP value for each sample; a color closer to purple indicates a larger value, while one closer to yellow indicates a smaller value. The more scattered the points in the graph, the greater the influence of the variable on the model. Time-dependent feature importance for the whole cohort and C/D AUC loss after permutation (**B**).

SHAP analysis was utilized to rank feature importance within the XGBoost model (Figure 4A), with RAR, CCI, SAPS II, APS III, and ABIC emerging as the top five predictors. Time-dependent permutation importance analysis identified RAR, ABIC, and BCR as the most influential factors for overall survival (Figure 4B). Partial dependence plots revealed nonlinear relationships between predictors and survival, with RAR showing the most pronounced negative impact on overall survival (Supplementary Materials, Figure S1).

#### 3.4.2. Local Explanations

The SurvSHAP(t) algorithm was applied to examine the time-dependent survival contributions of individual risk factors for selected patients. The y-axis represents SurvSHAP(t) values, where positive values indicate variables that increase overall survival probability, and negative values denote variables that decrease overall survival probability. Patient 1593, diagnosed with heart failure, was randomly selected from the survival cohort to illustrate the translation from population-level predictions to individual-level predictions. The analysis revealed that the variables APS III and SAPS II enhanced this patient’s survival probability, whereas RAR and BCR reduced it (Supplementary Materials, Figure S4C).

Although SurvLIME plots were conceptually similar to SurvSHAP(t), they provided distinct mechanistic insights into individualized risk factors. The left panel quantified the impact of covariates on survival probability, with larger shaded areas indicating stronger effects and higher local importance values correlating with decreased survival likelihood. The right panel compares model-predicted survival curves with SurvLIME-derived approximations; a closer alignment between the two functions validated the fidelity of the explanation. Upon reanalysis of Patient 1593 using XGBoost, SurvLIME identified serum chloride (detrimental) and pH (protective) as critical modifiers of prognosis (Supplementary Materials, Figure S4A). The strong concordance between SurvLIME-explained and model-predicted survival trajectories confirmed the accuracy of individualized risk estimation (Supplementary Materials, Figure S4B).

#### 3.4.3. Model Deployment

**Figure 5:**
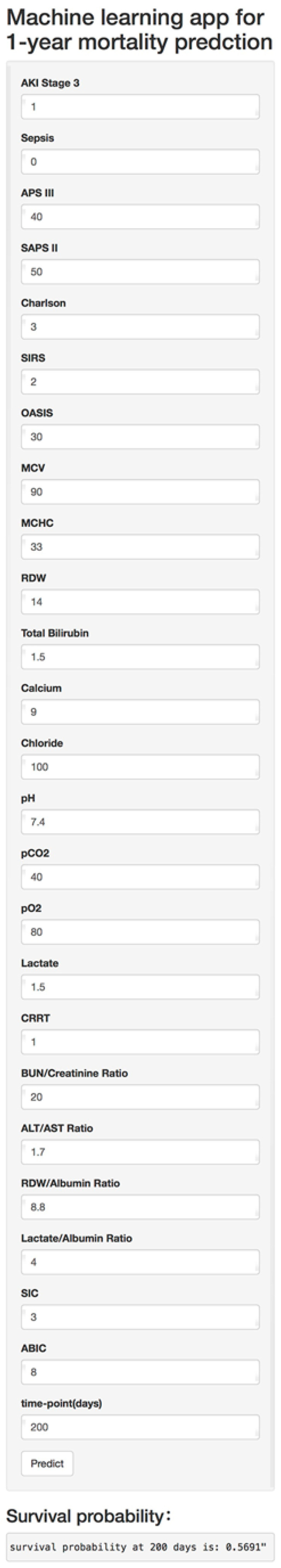
Online deployment interface: ICU congestive heart failure patient mortality risk prediction platform. Input patient clinical information and click the “Predict” button to obtain real-time, individualized in-hospital mortality risk assessment.

The web-based implementation of the XGBoost model was universally accessible to clinicians at https://cqmuwjy-app-for-mortality-prediction-app.shinyapps.io/deployment-1/, accessed on 25 January 2025. By inputting values for the 24 predefined clinical parameters, this tool automatically generated survival probability predictions for patients with HF admitted to the ICU at any time point within 365 days post-discharge (Figure 5).

## 4. Discussion

This study developed and validated an interpretable, time-dependent machine learning model based on the XGBoost algorithm to predict one-year all-cause mortality in ICU patients with HF. The model demonstrated superior predictive performance, with a C-index of 0.772, calibration as indicated by a Brier score of 0.165, and clinical net benefit across a threshold probability range of 30 to 100%. It was deployed online as a personalized risk prediction tool.

The SHFM and AHEAD scores, both used to predict mortality in heart failure patients, have shown suboptimal performance [5]. Similarly, the GWTG-HF model, when applied to assess risk in ICU patients with heart failure, yielded an AUC of 0.649 [19]. Consequently, existing risk scoring systems are neither specifically tailored to the ICU heart failure population nor exhibit outstanding prognostic performance. In contrast, ML has markedly improved predictive accuracy due to its ability to model nonlinear relationships and overcome multicollinearity. Adler et al., utilizing data from 5822 HF patients, demonstrated that ML algorithms enhanced predictive accuracy by 18 to 39% (AUC 0.88) compared to traditional risk scores (MAGGIC/AUC 0.74; ADHERE/AUC 0.63), with a 2.1-fold increase in sensitivity for identifying high-risk patients [20]. A systematic review by Shin et al., encompassing 686,842 patients, further suggested that in most studies focused on predicting readmission and mortality risk in HF patients, ML algorithms possessed superior discriminative capability relative to conventional statistical models [21]. In alignment with these findings, we assessed the long-term performance of four ML models (XGBoost, RSF, DeepSurv, and Cox regression) using 24 clinical features to predict mortality. We observed that the C-index of the XGBoost model was higher than that of the Cox regression model. This advantage could be attributed to several mechanisms: tree-based ensemble models; iteratively correct residuals through a boosting framework, thereby effectively capturing nonlinear interactions among high-dimensional clinical variables; and the integration of regularization techniques, such as L1 and L2 penalty terms (LASSO regression), which effectively addresses the multicollinearity challenges commonly encountered in traditional statistical models. In contrast to deep learning methodologies, which typically necessitate large sample sizes, XGBoost leverages parallel computation and pruning optimization to manage model complexity and mitigate overfitting risks within moderately sized medical datasets (*n* = 8960). This characteristic was corroborated in our study, where the DeepSurv model, despite its greater number of parameters, exhibited markedly inferior performance on the test set compared to XGBoost, indicating a tendency towards overfitting. The performance evaluation conducted on a relatively independent test set over a specified time span offers credible support for assessing the model’s generalization capabilities. Moreover, the deployment of the model within a network framework provided a distinct advantage for clinical translation and implementation, surpassing the capabilities of most existing studies.

Presently, the majority of machine learning studies simplify mortality to a binary endpoint, thereby forfeiting valuable temporal information. For instance, the study conducted by Tong et al. demonstrated that RSF and gradient boosting models, which utilized time-dependent analysis, significantly surpassed traditional binary classification models in predicting risk among heart failure patients [22]. These models exhibited superior short-term calibration and efficient utilization of variables, alongside a notable enhancement in the dynamic C-index. This research introduced an innovative application of time-dependent ML, achieving for the first time a continuous prediction of mortality risk in ICU patients with HF. In comparison to the in-hospital mortality model developed by Li et al. [23], our model represented a significant advancement in temporal resolution and clinical applicability. It offered a risk prediction platform capable of providing survival probabilities at any time point within a 365-day period, thereby offering a quantitative basis for dynamically adjusting treatment strategies.

This model significantly optimized feature engineering by incorporating multidimensional composite indices. Previous studies have suggested that an elevated RAR was associated with systemic inflammatory response, oxidative stress, and increased mortality risk [24]. The ABIC score, which incorporates liver and kidney function alongside coagulation abnormalities, has been validated in patients with coronary heart disease [25]. Our previous research demonstrated that ACAG was strongly positively correlated with mortality in HF and could enhance the predictive value of the SOFA and APS III scores [26]. The ALBI score was found to be independently associated with mortality in these HF patients, exhibiting an even more pronounced prognostic effect in younger patients and those with lower creatinine levels [27]. Additionally, a negative nonlinear relationship exists between ACR and mortality, which was challenging to adequately characterize using traditional Cox models [28]. These findings suggest that composite indices, by reflecting pathophysiological processes through a multi-organ interaction network, possess inherent nonlinear features and high-dimensional correlations that present opportunities for optimized ML. In the present study, we integrated six traditional prognostic scores (e.g., APS III, SAPS II) and eight composite laboratory indices (e.g., RAR, ABIC, ACAG). Notably, the contribution weights of RAR and ABIC in the model’s feature importance ranking surpassed those of the traditional scoring systems, indicating that composite laboratory indices—owing to their pathophysiological associations with organ dysfunction and systemic inflammatory response—may offer a more sensitive prognostic signal, thereby complementing previous studies.

The temporal validation set (2020–2022) encompassed the COVID-19 pandemic, a period marked by significant shifts in ICU practices and patient acuity. Despite these systemic disruptions, our model demonstrated stable performance (C-index = 0.772), suggesting that its dependence on composite biomarkers capturing systemic pathophysiology (e.g., RAR, ABIC) may buffer against transient clinical variability. However, the absence of explicit COVID-19 status annotations in the dataset precludes direct analysis of pandemic-specific effects on heart failure outcomes. Future work should investigate model performance in cohorts with confirmed SARS-CoV-2 co-infections to further validate its applicability in pandemic-influenced critical care settings.

Nonetheless, this study has certain limitations. First, the dataset contained missing values; however, multiple imputation methods were utilized to address these gaps, potentially approximating the true values. Second, determining whether HF was the primary cause of ICU admission and identifying the specific cause of patient mortality within the MIMIC database presented significant challenges. ICU admissions frequently resulted from critical conditions that impact multiple organ systems, including HF, which often led to multi-organ failure. From the patient’s perspective, all-cause mortality may serve as a more meaningful endpoint. Nonetheless, it was essential to acknowledge the distinct pathophysiological mechanisms that differentiate acute from chronic heart failure, as well as heart failure with reduced versus preserved left ventricular ejection fraction.

## 5. Conclusions

This study employed time-dependent ML techniques to develop an innovative risk stratification model for categorizing the one-year all-cause mortality risk among ICU patients with HF. The model is accessible through a freely available web-based calculator. This tool not only quantifies mortality risk but also unveils actionable pathophysiological insights. For instance, elevated RAR and ABIC scores may signal systemic inflammation and multi-organ dysfunction, urging clinicians to tailor therapies targeting these pathways (e.g., albumin supplementation, anti-inflammatory agents). Furthermore, the integration of SHAP/SurvSHAP(t) framework bridges ML interpretability with clinical reasoning, as demonstrated in recent bioinformatics advancements [29,30].

## Figures and Tables

**Table 1:** Optimal parameters of machine learning models in predicting one-year mortality.

Models	Optimal Parameters	
XGBoost	nrounds = 368, nthread = 1	subsample = 0.5488236
	eta = 0.004817722	colsample_bytree = 0.5026403
	max_depth = 8	lambda = 0.1330041
	min_child_weight = 4.4415141	alpha = 2.646525
RSF	num.trees = 264, mtry = 2	num.threads = 1
	min.node.size = 5	max.depth = 10
DeepSur	num_nodes = 246	batch_norm = TRUE
	learning_rate = 0.00111408	activation = “sigmoid”
	dropout = 0.3304397	optimizer = “adamax”

## Data Availability

The datasets presented in this study can be obtained from the MIMIC database on the premise of completing its training (https://physionet.org/content/mimiciv/3.1/, accessed on 24 November 2024).

## Supplementary Materials

The following supporting information can be downloaded at https://www.mdpi.com/article/10.3390/bioengineering12050511/s1, Supplementary Figure S1. The performance metrics of all models in the test dataset. Supplementary Figure S2. The calibration plots at the 365th day for all models in the test dataset. Calibration plots of predicted probabilities (X-axis) and actual proportions (Y-axis) for different prediction models. The diagonal dotted line represents perfect calibration. (A) DeepSurv model; (B) Cox proportional hazards model; (C) random survival forest (RSF) model; and (D) eXtreme Gradient Boosting (XGBoost) model. The XGBoost model from the machine learning algorithm obtained a fairly satisfactory calibration, while the other models calibrated poorly. Supplementary Figure S3. The decision curve analysis at the 365th day of all models in the test dataset, evaluating clinical utility. (A) Decision curves, plotting net benefit against threshold probability. Net benefit is calculated as (True Positives/n) − (False Positives/n) ∗ (Pt/(1 − Pt)), where Pt is the threshold probability and n is the total number of samples. (B) Net benefit of intervention avoidance curves, quantifying the net benefit of avoiding interventions based on model predictions. Supplementary Figure S4. The partial dependence plot (PDP) for the XGBoost model. It shows how the OS of the whole cohort changes if the value of one determinant is altered but all other factors are held constant. The y-axis represents the value of the survival function for each covariate. A wider area of the curve indicates that the greater the difference in levels of a factor, the greater the effect of that factor on OS. Supplementary Figure S5. The local explanations of all models, illustrating feature importance for individual predictions. (A and B) Local explanations provided by SurvLIME (Survival Local Interpretable Model-agnostic Explanations), showing the contribution of each feature to a specific prediction. (C) Local explanations provided by SurvSHAP(t) (Survival Shapley Additive Explanations (time-dependent)), showing the time-dependent feature attributions for individual predictions, quantifying how each feature influences the predicted survival function over time. Supplementary Table S1. Baseline characteristics of training data and test data.
