# Supplementary figures and images for: Development, Validation, and Deployment of a Time-Dependent Machine Learning Model for Predicting One-Year Mortality Risk in Critically Ill Patients with Heart Failure
Source: Bioengineering (Basel). 2025 May 12;12(5):511. doi: 10.3390/bioengineering12050511 (PMC12108603; doi:10.3390/bioengineering12050511)

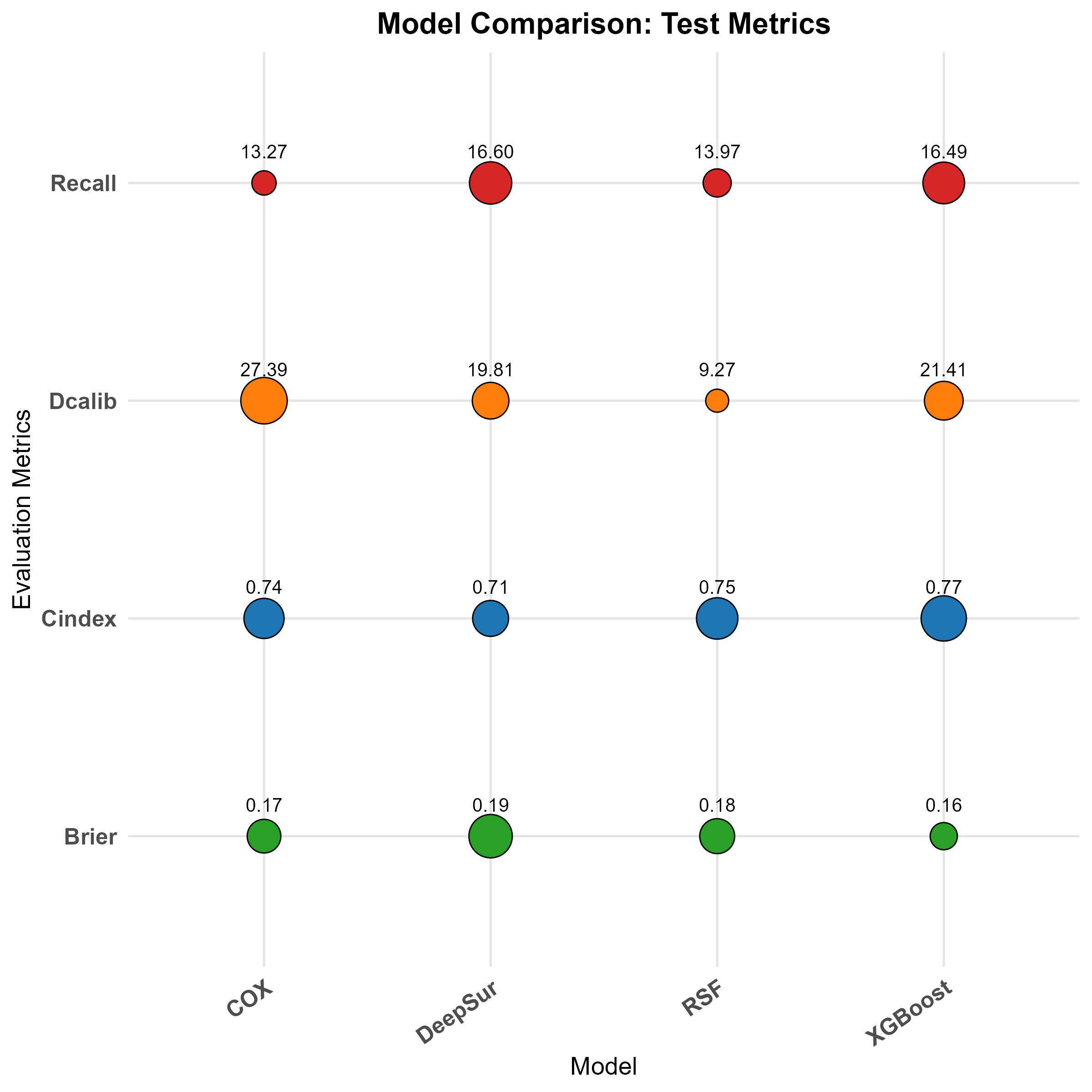

Supplement: Supplementary file 1 [file bioengineering-12-00511-s001.zip › Supplementary figure1.tiff]

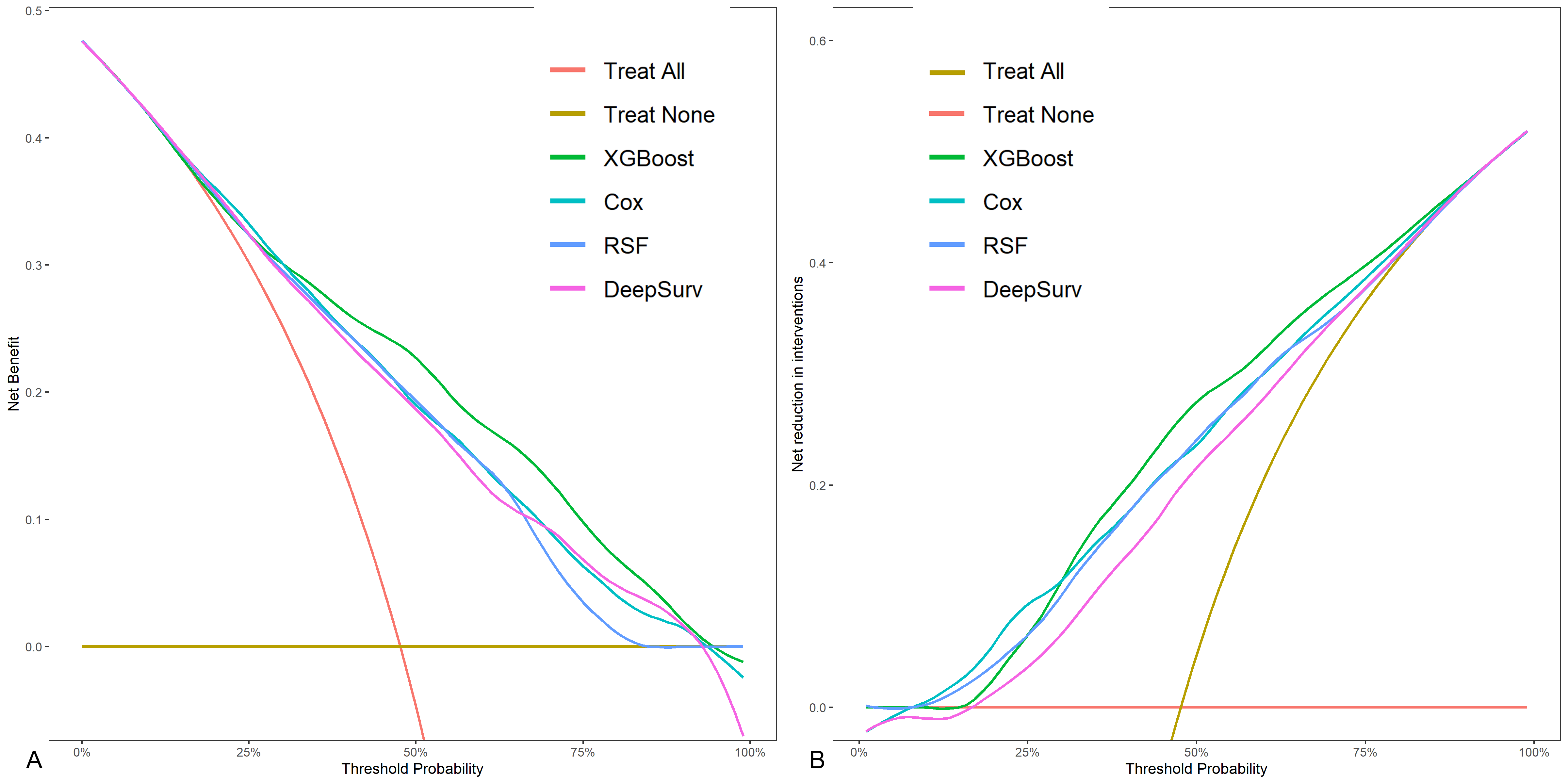

Supplement: Supplementary file 1 [file bioengineering-12-00511-s001.zip › Supplementary figure3.tif]
